# A Further Inquiry Concerning Constitutional Irritation, &c.

**Published:** 1837-04

**Authors:** 


THE
### BRITISH AND FOREIGN
### MEDICAL REVIEW,
FOR APRIL, 1837.

### PART FIRST.

### Analytical and Critical Reviews.

### Art. I.

1. *A Further Inquiry concerning Constitutional Irritation, &c.* By Benjamin Travers, f.r.s.
2. *Outlines of Human Pathology.* By Herbert Mayo, f.r.s.
3. *An Essay on Laryngismus Stridulus.* By Hugh Ley, m.d.
4. *Researches on the Diseases of the Brain.* By John Abercrombie, m.d.
5. *Lectures on the Nervous System.* By Marshall Hall, m.d.
6. *Papers on Diseases of the Brain,* (*Med.-Chir. Transactions,* vol. xix.) By J. Sims, m.d.
6. *Isis Revelata.* By J. C. Colquhoun, Esq.

In passing in review, in our last Number, the contents of the very valuable works of which the titles are prefixed, we considered at some length several of the most important points relating to the actual state of our knowledge of the physiology and pathology of the nervous system. After having discussed the fundamental physiological question as to the extent in which the nervous system is concerned in maintaining the organic life of animals, we endeavoured to make the reader acquainted with the present state of our knowledge of the pathology, firstly, of the nerves, and, secondly, of the spinal marrow and medulla oblongata. We shall now conclude the subject by endeavouring to ascertain the state of our knowledge as to the pathology of the brain itself, or those parts of the nervous system superior to the medulla oblongata.

When we attempt a survey of the state of our knowledge in regard to the Pathology of the Brain, we meet with a striking example of the difficulty which, in the present stage of medical science, attends every attempt to accommodate our pathological knowledge to nosological distinctions, and therefore to apply it in practice with confidence and precision. If we attempt to arrange cases of disease within the cranium according to their most prominent or urgent symptoms, we shall put together many which proceed not only from various external causes, but from various kinds of diseased action; and therefore, if fatal, present very various appearances on dissection. If, on the other hand, we give names to diseases only according to the morbid appearances which they leave behind them, the insurmountable practical objection immediately presents itself, that we shall have no names for many diseases, during the earlier and more important periods of their progress, and frequently none even until the anatomist's art shall have come to the assistance of the physician's diagnosis.

For example, how various are the causes from which headach, or coma, or convulsion, or delirium, or even palsy, or any combination of two or more of these symptoms may proceed; and, on the other hand, how various the series of symptoms which may attend either simple inflammation of the brain or its membranes, or the effusion of serum, or the rupture of a blood-vessel, or the growth of tubercles or of other tumours, or the chronic hardening or softening of portions of the brain!

Mr. Mayo is so well aware of this difficulty that, after describing the different " structural lesions" to which the different textures within the cranium are liable, he adds,

"The disorders of the cerebral functions are so far from having an exact relation to them, that, to convey a true idea of their character, it is necessary to give up the attempt to arrange them on anatomical principles, and to be satisfied with grouping them into natural families upon resemblance of symptoms alone; examining afterwards with how various conditions of the brain each disease may be associated." (*Outlines*, p. 182.)

This is an acknowledgment of the essential distinction between morbid anatomy and pathology, on which young practitioners will do well to reflect. It is not a short process of reasoning only, but a whole science that intervenes between the observation of morbid lesions, and the application of the knowledge thus acquired to the treatment of disease; and one of the most important subjects for investigation which this science comprehends, is the question, how similar lesions should be found in connexion with very various symptoms, and very various lesions with symptoms remarkably similar. In enquiries regarding diseases within the cranium, this question presents itself continually, and has invested the subject with a peculiar perplexity.

But we cannot exactly agree with Mr. Mayo as to the mode of arrangement of diseases of the brain, by which our pathological knowledge can be best brought to bear upon practice. In this, as in other cases, we think it plain that the notions attached to *genera* of disease must be made more comprehensive than has usually been the practice of physicians, and may thus be safely and advantageously connected with their pathology, as already ascertained; and that many of the terms which were formerly held to denote different diseases should be retained as the names of symptoms, or sets of symptoms, or diseased states, which may occur in the course of different diseases.

The general character of diseases should, we think, always be founded on the leading, and essential, and recognized distinctions of their pathology, (using that term in its most extensive sense,) and therefore should always be drawn up with special reference to their morbid anatomy. It is only by building on this foundation that we can be certain of forming a fixed and durable system of nosology, grounded, not on any arbitrary arrangements or accidental combinations of symptoms, but on the essential nature of the changes which constitute diseases. But, then, we must remember that each disease consists of a long series of changes, involving various functions of the body, and showing themselves by symptoms which vary in the progress of the case, and are modified by the circumstances of the patient; and that it is no reflection either on the character of the observer, or on the method which he follows, that he should often be unable, from seeing certain only of the changes that are going on, and these only during a portion,—often a very variable portion of their progress, —to give truly scientific names to many of the cases which he sees. We must remember, therefore, that certain symptoms, and sets of symptoms, are often invested with a practical importance hardly inferior to genera of disease, demanding and justifying the use of remedies, in many cases where it is still uncertain of what disease they form a part; although, in all cases, it is of the utmost importance to obtain this knowledge as soon as possible, in order to understand the danger that is impending, and to acquire confidence in the remedies that are truly indicated. Thus, headach, coma, convulsions, delirium, palsy, are not, properly speaking, diseases; but occurrences which may take place in the course of various diseases of the nervous system; their proper treatment must depend very much on the nature of the diseased action of which they form a part, and must therefore be very various in different cases; yet these symptoms are in many cases of themselves sufficient to justify important modes of practice, while the nature of the changes producing them is still obscure.

We must also remember that the distinctions of diseases (and particularly of those which are unconnected with the application of morbific poisons,) cannot be drawn with the same precision as those of animals or plants, or crystallized minerals, because in many cases two different diseases either graduate insensibly into one another, or give occasion to one another, and so constitute an unbroken succession of changes. Thus, we can have no doubt that many organic diseases of the brain are in reality effects of inflammation occurring there, especially if happening in unhealthy constitutions.

Entertaining these general views as to the arrangement of diseases, we cannot approve of the merely arbitrary arrangement of Mr. Mayo:—"Apoplexy, palsy, epilepsy, derangement, inflammatory disease, concussion, compression from injuries." In order to take a systematic view of the subject, the proper course, we apprehend, is this: first to study the various primary effects of external injury, whether concussion (previous to the accession of inflammation,) or compression from depressed bone or effused blood; and then the effects on the functions of the nervous system, of other external causes operating suddenly and violently, cold, heat, electricity, hemorrhage, and especially narcotic poisons. The works of Pott, Abernethy, Brodie, Travers, Kellie, Christison, and others, have illustrated this part of the subject nearly as completely as can be desired. Having thus acquired precise information as to the direct effects of these powerful external agents on the nervous system, we would next take Dr. Abercrombie as our guide in bringing into order the facts that are known as to its diseased states resulting from internal changes; and, so far as he goes, his arrangement appears excellent; but there are two important classes of nervous disease, which he has either omitted or only incidentally noticed, and of which we shall say a few words in the sequel.

Dr. Abercrombie's general division is into Inflammatory Affections of the Brain, Apoplectic Affections, and Organic Diseases of the Brain. By the term apoplectic affections, he understands all those serious diseases of this organ which take place *suddenly*, whether the affection be of the nature of coma or only palsy. It is obvious that these great genera of disease graduate insensibly, in some instances, into one another; yet the generic distinctions are both borne out by morbid anatomy, and generally applicable, and important to be applied in practice; they are simple, comprehensive, and important. We shall adopt his distinctions, although altering the arrangement of his classes.

I. Dr. Abercrombie has, we think, the merit of having observed, analysed, and arranged the very various symptoms of inflammatory disease within the head, more successfully than any preceding author. In so doing, we need hardly say that he has described and methodized the chief symptoms of the earlier stages of Hydrocephalus; that being the name given by the older authors to the only inflammatory disease of the brain and its membranes, which is of real and frequent occurrence, most commonly in children, but occasionally also in adults. That a very large proportion of strictly inflammatory cases where the brain is concerned should not, until lately, have been generally recognized as such, is sufficiently explained by the observation that, "from the rapid effects which all acute diseases of the brain produce on the sensorial functions, the patient generally becomes, at an early period, unable to express his feelings, and the proper symptoms of the disease are lost amid that suspension of all the faculties, to which we give the name of oppression of the brain:" i. e. the stupor, consequent even on the first stage of the inflammation, masks the symptoms which would otherwise have been recognized as strictly inflammatory.

It is true that, in many cases of fatal hydrocephalus, we find on dissection the effusion of serum only, without any decidedly inflammatory appearance; but, in many, such appearances on the membranes of the brain, (especially at its base,) or in its substance, are quite unequivocal: the symptoms in those which give this distinct evidence of their inflammatory nature are not to be distinguished from those of the cases where the effusion of serum only appears on dissection, and the experience of the juvantia and lædentia, in all the varieties of the disease in question, when it can be recognized in its early stage, abundantly confirms the conclusion of their inflammatory nature. The strictly and unequivocally inflammatory appearances often found in the brain and its membranes, in connexion with and sometimes without serous effusion, are reddening and hardening of portions of the cerebral matters, softening with discoloration of the same; effusion of false membranes, most generally seen between the pia mater and arachnoid; suppuration, circumscribed or diffused; and in a few rare cases ulceration, besides more chronic sequelæ, which will more properly be mentioned as organic lesions.

The very various symptoms observed in different cases in the commencement of a disease, which goes on, within a few weeks, to coma, convulsion, and the other concomitants of the last stage of hydrocephalus, and shews on dissection serous effusion in the ventricles, with or without these marks of inflammation, are arranged by Dr. Abercrombie as follows,—with the preliminary admission, in which we fully concur, that, for practical purposes, we cannot, as yet, depend on any distinctions of inflammation of the substance of the brain from inflammation of its membranes, and must be contented with a general "view of the symptoms which indicate inflammation of any of the parts within the cranium."

1. One set of cases are described as beginning with the phrenitis of systematic writers; "Fever, pervigilium, acute headach, impatience of light, suffusion of the eyes, and maniacal delirium." This combination of symptoms occurs symptomatically in a few cases of fevers and of mania; rarely idiopathically, excepting now and then from the abuse of strong liquors, and in hot climates from exposure to the intense heat of the sun. Sometimes also similar symptoms are seen from injuries of the head. There is reason to think that the inflammation in such cases is chiefly in the membranes and external surface of the hemispheres. When such cases are fatal, it is generally by rapid sinking of the vital powers; and the appearances on dissection are often slight.

On this form of the disease we would observe, that the cases of this description produced by insolation and by drinking, and we believe also some of those from concussion, are probably not strictly inflammatory; although they are cases in which a determination to the brain, chiefly affecting its external surface, and some serous effusion there may take place. Some such cases bear bleeding well, but in many the pulse soon becomes feeble, and full bleeding is certainly dangerous: and Dr. Abercrombie describes a set of cases which he has repeatedly seen, unconnected with the use of strong liquor, but in which the symptoms are just such as we see in many cases of delirium tremens; and the appearances on dissection, if they are fatal, and some of the remedies found useful, very similar to what is there seen;—cases of "remarkable restlessness, quickness and impatience of manner, obstinate watchfulness, and incessant rapid talking, the patient rambling from one subject to another with little connexion, but often without actual hallucination." (P. 6.) Such cases, he observes, are generally fatal, by sudden "sinking of the vital powers supervening on the high excitement, without coma," (p. 61;) just as happens in delirium tremens, the fatal event of which is very generally strictly in the way of syncope, not of coma. Such cases, when fatal, show very little morbid appearances; only "a very vascular state of the pia mater, with sometimes very slight effusion between it and the arachnoid;" appearances by no means unequivocal. "The disease is one of extreme danger, but does not in general admit of very active treatment. General bleeding is not well borne, and the treatment must in general be confined to topical bleeding, antimonials, purgatives, and the powerful application of cold to the head. This affection is most common in females of a delicate irritable habit." In his third edition, Dr. Abercrombie describes one case of this kind, and mentions having seen several others in which, "when the pulse became rapid and feeble, and the countenance expressive of exhaustion," stimulants in pretty full quantity, (e. g. a glass of wine every hour till the fourth time,) were decidedly beneficial. In this also there is the obvious analogy to the now established practice in delirium tremens; and, from what we have often seen of the effect of opium in similar circumstances in cases of that kind, we should judge it to be applicable here also, although not mentioned by our author.

We do not deny, that some of the fatal cases of this description may be cases of inflammation of the pia mater, fatal in its earliest stage, before the characteristic inflammatory exudations have appeared; but we know from experience that forty-eight hours or less of active inflammation of that membrane, on the external surface of the brain, are sufficient to produce a pretty thick sheet of lymph; and it may at least be inferred that the cases of real inflammation of the brain, where the symptoms correspond to the phrenitis of the older authors, are very rare.

2. Dr. Abercrombie describes a set of truly inflammatory cases, of great importance in practice, where "the first symptom that excites alarm," sometimes positively the first observed, "is a sudden attack of *convulsion*, generally long and severe," sometimes passing directly into fatal coma, in other cases returning repeatedly, with intervals of restoration of the senses. The pulse is very variable, and febrile symptoms occur. In some such cases, it appears distinctly from his researches, there has been inflammation of the substance of the brain, in others, of the membranes only; in some, fatal in its first stage; in others, not until much softening of the brain, or purulent effusion on its surface, had taken place. This appears evidently to be one of the most dangerous forms of the disease; but two unequivocal cases are given, (70 and 71,) in which full and early bleeding and purging were completely successful. There are cases, also, in which the first inflammatory symptoms are so slight as hardly to attract attention, and are observed by the early accession of coma, more or less profound, but with little or no convulsion. A striking example is given by Dr. M<sup>c</sup>Lagan, of Edinburgh, in the Edinburgh Journal for October, 1835.

3. The next form of the disease described is one more frequently seen, and, under early and active treatment, we believe, much more easily controlled. In this there are the usual febrile symptoms, sometimes ushered in by some shivering.

"The patient is oppressed and unwilling to be disturbed, and complains of *acute* pain in some part of the head, with flushing of the face and impatience of light. In many cases there is frequent vomiting for the first day or two; in others, this is absent. The pain is felt in various parts of the head, frequently extends along the neck, and sometimes to other parts of the body. The pupil is usually contracted, the eye morbidly sensible, sometimes suffused; the tongue generally white, but moist, sometimes quite clean. The sleep is disturbed by starting and frightful dreams, frequently with violent grinding of the teeth. The bowels are generally obstinate, but sometimes natural, and I have seen the disease attended throughout its whole course by a spontaneous diarrhœa. After some days, slight delirium begins to appear, &c., or, instead of delirium, a peculiar forgetfulness; the patient using one word instead of another, misnaming persons and things, &c. These symptoms are followed by a tendency to sleep, and this soon passes into coma. While these symptoms are going on, the pulse, which was at first frequent, usually falls to the natural standard, or below it."

It is unnecessary to proceed with this description; but, as every thing depends on recognizing this form of the inflammation of the brain, (somewhat chronic, but the most frequent form,) in its earliest stage, we shall hazard a little criticism on this epitome of the symptoms. We do not think the term *oppression* so applicable to the first stage of this disease as of continued fever. There is generally a peculiar aversion to motion, and especially to the erect position, which causes nausea and increases the pain; and often there is the impatience both of light and sound; but there is less general torpor of the sensorial functions than in the beginning of fever. We should say also, that the vomiting of almost all ingesta is more general and characteristic than our author's statement would imply; and that the pain of head, besides being more acute, is often marked by occasional twinges, or *stounds*, of peculiar intensity, causing the patient to scream out occasionally in a manner obviously different from the prolonged, drawling moan of typhus. The flushing of the face is often absent. The pulse has also in general, we think, a peculiar sharpness before it is reduced in frequency; and it is very important to observe that blood drawn from the arm is very often both sizy and contracted, and easily distinguished from the blood of incipient fevers, the crassamentum of which is comparatively flabby. We insist on these marks, because a good deal of experience has convinced us that, if this combination of symptoms is seen within the first two days, and met by full and repeated bloodletting (from the arm, if the patient is above three years of age,) and free purging, the greater number of such cases will do well, and the convalescence be so rapid and complete, as to indicate unequivocally that an inflammatory disease had been cut short. In general, the more violent the pain, and the more distinctly this combination of symptoms is observed, the more quickly and surely will the early depletions succeed.

4. In the next form of the disease, the symptoms advance more slowly and are more insidious, and more like continued fever; the headach slight, or soon abating; with impaired appetite, disturbed sleep, general uneasiness, pulse under one hundred. "Amid remissions and aggravations, eight or ten days may pass before the disease assumes a decided character." An attentive observer may remark that the headach, though not severe, is more constant than in other febrile diseases, and continues, *with a peculiar unwillingness to be disturbed*, when the tongue is cleaning, the pulse coming down, and the appetite returning. " It may not be till the twelfth or fourteenth day that the pulse suddenly falls to the natural standard, or below it, while the headach is increased, with tendency to stupor; and then the usual symptoms of the later stages follow. This form," as Dr. Abercrombie justly observes, "is worthy of a separate description, on account of its insidious character in the early stages, and its frequent occurrence. Cases occur in which there is still less appearance of affection of the head, or even not the slightest complaint of headach throughout the whole disease." The more obscure and insidious the symptoms, in general is the disease more intractable.

5. The last form of the disease described by our author, and chiefly seen in adults, is where there is at first violent headach without fever, the pulse at or below the natural standard from the first; but the pain acute and shooting through the head, with unwillingness to be disturbed, often vomiting, and then early delirium.

We may quote here, before entering on some pathological questions connected with inflammation within the cranium, Dr. Abercrombie's general and most judicious observation on the treatment, that

"The remedies are few and simple, but every thing depends on the use of these being adopted at an early period, and in the most decided manner. Those on which we chiefly rely are bloodletting (general and topical), active purgatives, and cold applications to the head. Benefit is also derived from antimonials, and, in some states of the disease, from digitalis. The effect of blistering in the early stages is ambiguous." . . . "In those cases which assume the more acute or active forms, general bloodletting must be used in the most decided manner; while, in the cases which assume the more chronic character, as in many of the common cases of hydrocephalus, it has less control over the disease, and is not borne to the same extent."

We must allow that this last observation is true, yet we think there is much practical risk that it may be allowed to bias the minds of young practitioners too much. In many cases we believe that bloodletting is ineffectual, only because the disease is not recognized, at least with such confidence as to ensure its energetic employment in the early stage. When the disease is well marked, even although its progress may have been rather slow, we have so often seen that a certain degree of effect is produced on it by bloodletting, and have witnessed so often the utter inefficacy of all other remedies without it, that we can never think ourselves justified in declining to urge it as far as can be safely done.

Dr. Abercrombie's observations on the importance of active purging is, we think, expressed in too unqualified a form.

"This," he says, "is the remedy from which, in all forms of the disease, we find the most satisfactory results; and, although bloodletting is never to be neglected in the earlier stages, my own experience is that more recoveries take place from head affections of the most alarming aspect, under the use of very strong purging, than under any other mode of treatment. In most of these cases, indeed, full and repeated bleeding had been previously employed, but without any apparent effect in arresting the symptoms. The most convenient medicine for the purpose is the croton oil."

Now, although we believe it to be generally true that more recoveries from *bad symptoms* of affection of the brain take place under the use of purgatives than of bloodletting, yet we are confident that a greater number of recoveries from inflammation of the brain take place within a very short time after, and in consequence of, bloodletting; but then these are cases treated in the early stage, *before the symptoms have become bad.* The time when bloodletting can be expected to be decidedly useful is generally at an end when coma has supervened: a few cases recover, chiefly under blistering and purging, and probably under the use of mercury, at a more advanced period; but the proportion of these to the deaths is small, in comparison with the proportion of recoveries from the early stage, where the most powerful remedy used was full bloodletting: and, although there are cases in which no good effect appears at the time from bloodletting, and the symptoms abate afterwards under purgatives, it can hardly be doubted that the former remedy had most materially aided the effect of the latter. It is certain that, when blood is freely drawn in the beginning of the disease, the subsequent effect of purgatives is manifestly increased; so that in such cases the peculiar torpor of the bowels, regarded by some as characteristic of hydrocephalus, is rarely to be seen.

On the use of mercury Dr. Abercrombie gives an opinion which many of our brethren in this part of the island may regard as heterodox, but which agrees perfectly well with our own observations,—viz. "that it is useful chiefly as a purgative;" and that, if used in the early and more distinctly inflammatory stage, so as to affect the mouth, it may be injurious. Formerly it was given "to promote the absorption of the effused fluid, then supposed to be the essence of the disease; it is now given to correct the biliary secretion and the functions of the digestive organs, which, according to certain modern doctrines, hold so prominent a place in almost every class of diseases. In affections of the brain, as in other diseases, it is highly proper that these secretions should be attended to, but it is not thus that we are to expect to cure hydrocephalus." In another place he justly observes, that "every practitioner who divests himself of system, and attends to what is passing before him, will find that hydrocephalus runs its course with every possible variety in the appearance of the evacuations, and that, even at the most advanced periods of the disease, they may often be found to be perfectly natural." To this we may add that, although many practitioners, both in England and on the continent, believe in a peculiar efficacy of mercury in checking inflammations in various membranes; yet, if they were always careful to distinguish what is due to mercury as a purgative, from what is due to it as a specific affecting the mouth,—and what is truly its effect, from what is the effect of other remedies used at the same time,—we strongly suspect they would find their belief to have little foundation in personal observation. If there be any virtue in the specific agency of mercury, we should say, from any thing we have seen, that it is in the late stage of the disease, when effusion may be suspected, and when the comatose tendency is strong. In a few such cases, of slower progress than usual, we believe it is really efficacious.

Dr. Abercrombie's enumeration of the *causes* of inflammation within the brain is, we think, somewhat defective. He mentions particularly its frequent occurrence in *the course* of other febrile diseases,—as continued fever, scarlatina, measles, &c.: we think he should rather have said, immediately after the decline of these diseases; and then he might have added, that, in the same circumstances, other inflammations (e. g. of the pleura and of the kidneys,) are easily produced—all of them, we believe, most generally by slight exposure to cold—and are insidious and dangerous. He mentions particularly the case, by no means uncommon, of children, convalescent from scarlatina, "seized, perhaps after some exposure to cold, with headach, which after a short time is followed by convulsion, and then by blindness and coma." These symptoms, he says, "are apt to be ascribed to sudden effusion in the brain, but the disease is entirely inflammatory, and the patient can only be saved by the most vigorous treatment, by bloodletting, purgatives, and other similar remedies. Upon this plan many such cases recover." To this we have only to add, that in many such cases it will be found, on examination, that the urine is of low specific gravity and coagulable, indicating an inflammatory disposition in the kidneys, and, although not modifying the practice, assisting to explain the rapid accession of coma.

He mentions particularly, as causes of this inflammation, injuries of the head, even although apparently slight; suppressed evacuations, particularly of the menses; he gives cases illustrating its "frequent extension from disease of the ear, and connexion with previous diseases of a chronic or scrofulous character in other parts;" he barely notices "passions of the mind, stimulating liquors," &c. as its exciting causes, and more particularly insists on "exposure to the intense heat of the sun." We have seen several cases of children in which a sudden fright has acted most distinctly as the exciting cause of the disease; but we have our doubts as to the strictly inflammatory nature of the affection of the brain, produced either by drinking or by insolation exclusively. In the case which he gives in illustration of the last, of a young man who had bathed *twice* in the river Tweed in a hot day, and, "on coming out the second time, lay down on the bank, and fell asleep without his hat, exposed to the sun," awakened speechless, and in whom inflammation and suppuration were found ultimately to have taken place in the brain; it is obvious that the effect of the heat on the circulation in the head, in accordance with the luminous views of Dr. Currie on cold as a cause of disease, would be aided by chilling and depressed circulation in other parts of the surface, and particularly in the extremities. And we think it should be more distinctly stated, that exposure to cold is a very frequent exciting cause of this, as well as of other internal inflammations; in proof of which we may state that we have observed, on examining the records of a numerously attended dispensary, that the numbers of cases of hydrocephalus increased in the cold seasons of the year, fully as distinctly as those of inflammations within the chest.

Reverting to the pathology of cases of this description, we have to notice three questions, to which Dr. Abercrombie and Dr. Sims have particularly addressed themselves:—1. The nature of the connexion of the latter symptoms of the disease and death of the patient with the serous effusion in the brain, hardly requires so much attention now as at the time when Dr. Abercrombie first wrote on the subject. It is now generally known, to use his own words, "that there are many facts on record showing the presence of fluid in the brain in large quantity, without any alarming symptom having resulted from it; that it is not the mere presence of a certain quantity of fluid in the brain that gives rise to the symptoms of hydrocephalus, and that a disease may go through all the usual symptoms of hydrocephalus, and terminate fatally, without any effusion; that the prominent symptoms, therefore, are not the result of the effusion, but of that disease of the brain of which effusion is one of the terminations, and that this disease is of an inflammatory nature." Yet this last observation, we think, requires some limitation, and particularly when it is afterwards stated that "slowness of pulse, followed by frequency, squinting, double vision, dilated pupils, paralytic symptoms, and perfect coma, may exist in connexion with a state of the brain which is active or simply inflammatory;" although we may admit that in a few cases this is strictly true, yet we must remember that, in a great majority of cases, these symptoms of the last stage of hydrocephalus are in connexion, not with active inflammation, but with the lesions resulting from inflammation, and already effected; and that, when they appear, the time for bloodletting is generally over. They are the result of organic injury done to the substance and intimate structure of the brain, and more especially done, *through the substance of the brain, to the upper part of the spinal cord;* and of that injury, we think, it cannot be doubted that effusion of serum, *rapidly effected,* is a frequent, probably the most frequent, immediate cause. That a *very slow, gradual* effusion should have no such effect, is a fact strictly in consonance with what is observed as to the growth of tumours, or other causes which gradually compress, alter the form, or even displace portions of the brain or nervous system, without materially injuring their functions. That the very same symptom should result from disorganization of the cerebral substance, or even from the pressure of blood congested within its vessels, is no proof that serum, where it has been rapidly effused, and presses, as it must then do, in an unusual degree on the healthy brain around it, may not be an adequate cause of the symptoms of the last stage of these very numerous cases where it constitutes a material part of the appearances seen on dissection.*

These observations apply likewise to the statements of Dr. Sims on this subject, which, although highly satisfactory, as showing that much serous effusion may take place in the brain without coma,—indeed almost without any symptom,—would be too much relied on, if they were thought to indicate that serous effusion is in no case an adequate cause of coma. Dr. Sims gives fifty cases of death from diseases not affecting the head, in forty-five of which more or less morbid effusion of serum was found in the brain, "excluding," he says, "all cases in which coma, convulsion, or other known symptom of cerebral disease had occurred during life;" four of serous effusion along with old apoplectic cysts, where the patients were destroyed by diseases not cerebral: and six of similar serous effusion, of unquestionably long standing, with old apoplectic cysts, where the patients were destroyed by recent extravasation of blood within the cranium." On looking over these cases, we confess we are not satisfied with the evidence, either that the effusion had in all been of long standing, or that it was in all unconnected with the symptoms recently preceding death. He gives also various cases of simple sanguineous apoplexy, where there was turgescence of the vessels of the brain after death, (an appearance on which, we apprehend, he relies rather too confidently as indicating the state of the parts before death,) but no effusion. But all these cases do not prove that, when serum is *rapidly* effused within the brain or on its surface, it may not be a cause of urgent symptoms, and even of coma, advancing so rapidly as to cause the disease to be set down as apoplexy; they do not therefore justify the conclusion that "most of the cases of reputed serous apoplexy (i. e. where effusion of serum is found on dissection,) are referrible to simple sanguineous apoplexy," (i. e. to congestion of blood within the vessels, supervening on old and harmless effusion from them;) they only establish the possibility of such an occurrence. It is fair to observe, however, that Dr. Sims is not "prepared to state that no such disease as serous apoplexy exists." Indeed, if it be admitted that mere increased determination of blood, without effusion, may so injure the texture of the brain as to cause rapidly advancing coma, it seems to follow, *a fortiori*, that such increased determination, with effusion, may do the same. Accordingly, while, in some of the cases recorded by him as "cases of serous effusion of old standing," where death had taken place in the way of coma, and which,

* We once saw a case in which (not by our advice) more than an ounce of serum had been removed by paracentesis from the ventricles of the brain of a child in the last stage of hydrocephalus, without yielding of the sutures. The effects were, a slight diminution of the coma, a decided reduction of the frequency of the pulse (by more than twenty beats in the minute), and an equally obvious improvement of its strength, and probably a protraction of life for a day or two. Of course, the whole fluid could not be evacuated, and it was very quickly replaced, so that the ventricles were distended when the head was examined after death; but the evidence was clear as to part of the symptoms having been the direct effect of the effusion.

he says, "are frequently termed serous apoplexy, but are more properly referrible to simple sanguineous apoplexy," his statement of the pathology may be correct; there are others (particularly Cases 20 and 21,) in which we think it much more probable that a great part of the effusion found on dissection, had taken place during the continuance of the stupor, drowsiness, delirium, &c., which lasted, in the one case two days, and in the other eight days, before death.

Notwithstanding, therefore, the objections stated both by Dr. Abercrombie and Dr. Sims to the term "serous apoplexy," we cannot help thinking it, both pathologically and practically, the best name for a class of cases of frequent occurrence in advanced life, where the chief symptoms are merely loss of recollection, more or less general, and drowsiness, more or less bordering on coma, with some headach perhaps, but with little or no fever and no palsy, gradually increasing, and fatal after a few days or weeks, and where serous effusion is the chief appearance on dissection. That it is impossible to distinguish such cases with certainty during life, from some of the cases of organic disease of the brain, and that no peculiarity of practice is justified by this opinion of their nature, we willingly admit; but, in fact, the whole pathology of these cases is very much akin to that of cases of "perverted nutrition" within the head.

There is a question regarding serous effusion within the head, on which neither of these authors has touched, but which is probably of some consequence in reference to the variety of symptoms observed in cases where this effusion may be found,—viz. that in some cases the morbid effusion is nearly equally distributed beneath the arachnoid, on the surface of the brain, and in the ventricles; the communication at the posterior end of the fourth ventricle, so particularly noticed by Magendie, being as open as in the natural state; whereas in others this communication is closed, (sometimes by deposits of lymph, sometimes, as it appears to us, merely by the great distention of the lateral portions of the fourth ventricle,) and the surface of the brain is preternaturally dry. The former is the case in the effusions found after fever and after delirium tremens, and likewise (as Dr. Sims's paper shows,) in many of the effusions which take place very gradually in old persons, and are attended, during the greater part of their progress, by no symptoms of diseased brain. But the latter is by far the most common case when the symptoms have run the usual course of hydrocephalus, and when the other appearances are more decidedly inflammatory.

2. There has been some difficulty about the proper view to be taken of softening or "ramollissement" of the brain. Dr. Abercrombie restricts this name to the case of partial softening, without change of colour, giving a few cases only of the "ramollissement jaune" as undefined suppuration, and of the "ramollissement rouge" as inflammation fatal in the first stage. The softening, without discoloration, he was at first disposed to consider as a direct effect of inflammation, often found in connexion with, or even graduating by insensible degrees into unequivocal effects of recent inflammation; but, after considering the statements of Rostan and other French pathologists, he so far modified that opinion, as to admit of another variety of it, not strictly inflammatory, but analogous to the gangrene which results from diseased arteries and impeded circulation; occurring chiefly therefore in old persons, generally in connexion with apoplectic effusions, and falling peculiarly under the notice of M. Rostan. We have little doubt that he is right in the belief that the lesion in question may depend on both these causes; but, in some cases, we have seen it surrounding apoplectic cysts or in contact with serous effusions, where neither marks of active inflammation nor disease of the arteries was discernible, and where we would regard it therefore merely as a perversion of nutrition, resulting apparently from mechanical injury rather than decided inflammation of the brain. When a recent clot of blood is found in the interior of a softened portion of the brain, it may have happened either that the coagulum effused caused the inflammation and softening, or the disorganization of the brain led to the effusion of blood, and the course of the symptoms only can decide. In some cases, the cause of this lesion is quite obscure. Its symptoms are equally uncertain, although generally it may be regarded as the accompaniment of coma and spasms, rather than of the earlier inflammatory symptoms. The permanent rigidity of the limbs, stated by the French pathologists as nearly a pathognomonic sign, is only a frequent accompaniment of it, and may result, as we have ourselves seen, from nearly an opposite state of the brain. A transient state of tonic constriction of the limbs is common, and often recovered from in fevers. (*Abercrombie*, 109.) In four of the cases mentioned by Dr. Sims, when, after death, ramollissement was found to exist, there was no rigidity; in one only did this symptom occur, and in this a previous state of softening appears to have been cured.

Dr. Sims has applied himself to the question whether this change of texture of the brain is susceptible of cure, and answers it in the affirmative, trusting entirely to an examination of morbid appearances in old cases of diseased brain. His attention seems to have been directed to the red ramollissement, and the softening without change of colour in the white parts of the brain. We have our doubts whether he is correct in the belief that all the appearances he describes were truly the marks of previously softened brain, but other appearances which he has mentioned seem to us satisfactory indications of ramollissement in the process of cure. When we hesitate in arriving at the same conclusion as Dr. Sims, it is rather because the subject appears to require farther investigation, than because we think that he has speculated unfairly on the facts which he has collected. The conclusions at which Dr. Sims has arrived respecting the curability of ramollissement of the brain can scarcely be regarded as applicable to all the forms which that disease assumes. He does not notice that form of which Dr. Abercrombie speaks, and which has much attracted the attention of the French pathologists, where the "cerebral mass is broken down into a soft pulpy mass like thick cream or custard *retaining its natural colour*, but having lost its cohesion and consistence. It may be found in *any part* of the brain." But we will proceed to notice the signs of cure, and the forms of ramollissement which Dr. Sims has commented on, in his own words.

"The traces of cure of ramollissement of the grey matter are, absorption of one or more layers of this substance on the convolutions, and adhesion of the pia mater to the part; *holes in the grey matter of the corpora striata and other central parts, together with atrophy and flattening.* When transudation from the blood-vessels, or extravasation, has taken place, constituting red ramollissement in the grey matter, a permanent fawn colour of the atrophied convolutions, and of the small holes in the other parts, is observed. The slightest form of this softening of the grey matter is noticed in the case of purpura hæmorrhagica; in others, we have one or more layers removed, or the entire grey matter, leaving the white matter of the hemisphere visible. We sometimes see merely *small holes in the corpora striata;* at others, cavities of various sizes and forms, with a marked wasting of these bodies. The traces of the cure of ramollissement in the white matter are, the numerous clean or scooped-out holes containing a limpid fluid, some of which are observed to be lined by a fine transparent membrane, *others appear as if worm-eaten.* These holes are of various sizes and forms, from minute points to the magnitude of a bean; *the porous cheese or new-bread appearance;* the hardened state of the white matter generally in these brains, and particularly in the parts contiguous to the holes; the granular state of the white matter indicating cicatrices, the hardened state of the corpus callosum, fornix, &c., found in the brains of children and young persons, with fluid in the ventricles; probably the consequence of previous inflammatory ramollissement, at an earlier period of life. Where there is observed the fawn-coloured deposit in these holes of the white matter, they are traces of red ramollissement of the white matter, or probably, in some instances, of what has been sometimes termed capillary apoplexy."

The appearances, the description of which we have given in italics, Dr. Sims has fairly shown to be proofs of ramollissement in process of cure. Cases 1 and 4 strongly illustrate this fact. The worm-eaten and bread-like appearance are here found surrounding cavities lined by a fawn-coloured membrane, or containing shreds of brown filamentous tissue and fluid, (i. e. apoplectic cysts.) We do not, however, think that the holes which are spoken of as clean and scooped out, are shown to be the effects of the cure of softening of the brain, and indeed, of the instance which Dr. Sims has given of these appearances, (Case 5,) he only says that it is "highly probable" that such is the case. The cases which are adduced as evidence of absorption of the grey matter being a result of the cure of ramollissement, are not quite conclusive. There appears to be much reason to believe, that, in Case 6, where a large fawn-coloured deposit occupied the space effected by the absorption of the grey matter of the surface, in some places down to the white matter, this absorption was owing to the pressure from effused blood. A similar and extensive extravasation of the same colour existed between the dura mater and arachnoid, and the brain was atrophied. Cases 7, 8, may also be similarly regarded; in neither of which is there any evidence of ramollissement. That the extravasations there mentioned may *in some instances* be either cause or effect of softening of the brain, there is reason to believe; but there does not appear to have been any change in the cerebral substance, and the only appearances spoken of are those presented by the effused matter itself. But perhaps this is explicable on the view which Dr. Sims takes of the nature of the red ramollissement, as seen in the above quotation, where he speaks of "transudation from blood-vessels, or extravasation," as "constituting red ramollissement in the grey matter." This is an extension of the application of the term which we are not disposed to admit, any more than that such effects necessarily accompany the changes of colour and consistence in other congested or inflamed parts. The extravasation we should regard as something superadded to the red ramollissement, and not necessary to constitute it. Indeed, in a previous part of his paper, Dr. Sims speaks of the colour of red ramollissement as arising from dilatation of the capillaries carrying red blood. (P. 382.)

But that in some instances absorption is an evidence of the cure of softening, we think that Dr. Sims has shown in Cases 9 and 10. In the former, a portion of brain was absorbed, and other portions of grey softened matter adhered to the dura mater at the part; in the latter, the absorption was associated with several small holes, and with ramollissement in an early stage; the symptoms during life having corresponded to the various stages of disease found after death. The hardening which is sometimes found in the parts contiguous to the small worm-eaten holes would also appear to be a part of the curative process of ramollissement; and of a granular structure associated in Case 12, with small holes, Dr. Sims offers the very probable explanation, that it resulted from the coalescence or adhesion of some of these small cavities.

3. In regard to the connexion of inflammation with tubercular disease in the brain, Dr. Abercrombie gives several cases, (81 to 89,) in which tubercles in their advanced stage were found in different parts of the brain and cerebellum, in patients who had previously had some chronic head symptoms, but in whom the symptoms immediately preceding death were those of hydrocephalus, and in all but one, the ventricles were found distended with serum. These were obviously cases in which the tubercles had acted as a permanent predisposing cause of inflammatory action and serous effusion, but give no information touching the original mode of production of the tubercles themselves, farther than that the symptoms attending their original deposition were generally obscure, and that they often coexisted with numerous tubercles in other parts of the body. Dr. Abercrombie observes, however, that "there is reason to believe that the *deposition* of tubercular matters in the brain, as in other parts of the body, is often the result of inflammatory action of a low scrofulous character; that it may at first be excited by injuries or other causes of inflammation, and may then advance gradually in a slow insidious manner;" and he gives two cases in illustration of this, where there was strong reason to ascribe the commencement of tubercles in the brain to external injury. But he does not seem to have observed what we have seen so frequently as to be able to state it as a common occurrence, tubercles *in their first stage* on the pia mater, consisting of roundish nodules of semi-transparent lymph, set on an inflamed base and in connexion both with effusion into the ventricles and with unequivocal indications of recent inflammation; flakes of soft lymph, or "ramollissement rouge," in their own immediate neighbourhood. Such cases we regard as the clearest proofs of the principle which he has here stated, and as satisfactory evidence that in *some* cases of threatening tubercular disease, particularly when the peculiar diathesis is least strong, the timely use of the antiphlogistic remedies may prevent their formation or check their growth, and perhaps sometimes determine their absorption.

II. This subject naturally leads us to consider the strictly Organic Diseases of the Brain and Cerebellum. Indeed, the connexion of these with the inflammatory diseases is obvious and intimate. Other adventitious textures, besides tubercles, are probably often connected with inflammatory action in their commencement, and often act as a predisponent cause of it in their progress; and there is often no possibility, in this as in other cases of internal disease, of distinguishing, during life, the symptoms of simple chronic inflammation and its consequences, from those which result from the deposition "des matières hétérologues" in the viscera; and therefore we think that the subject of organic disease ought to have been placed by Dr. Abercrombie next to that which we have now considered.

Of the very various symptoms which experience shows to be indications, in different cases, of organic disease of the brain, Dr. Abercrombie makes the following judicious arrangement:

1. In one class of cases, the only prominent symptom during the time when the disease must be making its chief progress is *headach,* long continued and severe, but often remitting remarkably, and even regularly. The diagnosis of this headach is often difficult, particularly as the stomach is often disordered; but the intensity of the pain bears no proportion to the degree of the dyspeptic symptoms, and the increase of the headach from excitement or exertion is often characteristic, especially if the patient was not previously of irritable habit. The face is generally pale, the pulse feeble, and depletory remedies ineffectual. Such cases, and indeed all cases of organic disease in the brain, are exceedingly various in their duration and progress, and may be fatal in various ways, either by inflammatory action and effusion supervening, or by coma or convulsion, or by gradual exhaustion, without serous effusion.

2. In another class, after some continuance of headach, the nature of the disease shows itself by *some affection of the senses* or of the *intellect.* Here, of course, a certain number of cases of amaurosis is included.

3. In another class, there are not only pain of head and some affection of the senses, but repeated attacks of *convulsion*, either in occasional paroxysms without other symptoms, (constituting one frequent variety of epilepsy,) or in repeated paroxysms within a short time, and with febrile and inflammatory symptoms.

4. The next class comprehends other cases of epilepsy, where there are repeated *convulsions without any affection of the senses*, and often with very little pain, in the intervals; the memory being, however, impaired sometimes for a space after each paroxysm, and in general more permanently after the disease has lasted some time.

5. In the next class the most prominent symptom is *palsy*, more or less general, but coming on gradually, with which there is often pain in the palsied limbs; and more or less of the symptoms already enumerated may likewise be present.

6. Another important class of these cases present no obvious affection of the head during the greater part of their course: the symptoms are *referred to the stomach;* and *sickness and vomiting*, sometimes at pretty regular periods, often irregularly, but generally in sudden paroxysms, are the chief symptoms. There is no mystery in this to those who reflect that vomiting is not an affection of the stomach, but an affection of various muscles, excited through the spinal cord by a sensation which probably resides at the medulla oblongata; and which sensation may be produced, no doubt, by certain changes at the stomach, but likewise remarkably by changes at various other parts, and, among others, by changes in the brain, as in sea-sickness and in many cases of concussion. These cases are sometimes attended with headach, and generally, after a time, with fits of delirium or of loss of recollection.

7. The last class which he describes consists of cases where there are

*many slight and transient apoplectic attacks;* sometimes mere giddiness, sometimes transient loss of voluntary power or of recollection, or absolute coma. With these some of the permanent affections of the senses may or may not be combined. Frequently the fits of giddiness and loss of recollection are brought on by exertion, and relieved merely by rest. We are somewhat surprised that our author did not add to this enumeration of the forms of disease which *may* be connected with organic disease of the brain, the numerous modifications of *partial insanity.*

Now, in connexion with any one of these various sets of symptoms, there may be any one of a considerable variety of organic lesions within the head,—tumours formed by thickening of the membranes or by deposition of lymph between their laminæ; indurations or chronic softening of portions of the cerebral matters, (all which lesions may be ascribed to mere chronic inflammation;) ossifications of the membranes, or exostoses projecting from the inner table of the skull; tubercles; encysted tumours, containing an albuminous matter; dense tumours of whitish or reddish matter, often growing from the membranes, sometimes imbedded in the substance of the brain; serous cysts, and true hydatids.

Dr. Sims has described numerous cases, and referred to others described by previous authors, illustrating the *hypertrophy* and *atrophy* of the brain; the latter of which appears to be commonly the result of the general wasting from age, of extraneous pressure, or of emaciating diseases. Serous fluids, and deposits of bone on the skull, occupy the space which is left by the diminished brain. This bony deposit is frequent on the internal surface of the cranium, but it sometimes occurs within the diploë. To this change is ascribed, with much probability, "the blunted feelings, loss of memory, diminished mental powers, feeble movements, muscular tremors, and probably impaired energies in the actions of the thoracic and abdominal viscera."

The hypertrophy is certainly to be regarded as a peculiar form of organic disease, consisting in increased, and likewise, to a certain degree, in perverted nutrition, and marked not only by increased bulk of the substance of the brain, (sometimes, in children, leading to great enlargement of the size of the head,) but likewise, in many cases, by flattening of the convolutions, and almost complete obliteration both of the divisions of the convolutions and of the cavity of the ventricles, the surface of the brain being remarkably dry, and its substance usually firm, white, and nearly bloodless. The hypertrophy is sometimes limited to parts of the cerebrum, and it does not appear to affect the cerebellum. Dr. Sims particularly presses the importance of noticing this change, (and, from the fact of its having been little noticed by British pathologists, it would appear that it has frequently been overlooked, rendering such a caution necessary,) from its obvious bearing on the practical treatment of apoplexy and other diseases of the brain. The condition appears to be often connected both with inflammatory attacks and with apoplectic seizures. It is probable that by it apoplexy is more rapidly induced, and that life may be destroyed by a very small clot of extravasated blood. When the firmness and apparent compression of the brain are found, the symptoms generally appear to have been more acute than when the texture of the brain has been more natural. But certain cases of hypertrophy, even with some change of texture, seem to have been without obvious symptoms. A practical deduction of much importance is strongly illustrated by Case 1, where the operation of tapping the skull was recommended, on the suspicion of hydrocephalus, but where, after death, the brain was found to be simply hypertrophied. But the means of diagnosticating this affection remain a subject for future investigation. We subjoin an abstract of a very elaborate table, containing the result of an examination instituted to ascertain the average weight of the healthy brain. The result very nearly corresponds with that of Sir William Hamilton; Dr. Sims estimating by avoirdupois, and Hamilton by troy weight.

| Years. | | Number weighed. | | Average. | |
|---|---|---|---|---|---|
| 1 to 2 | — | 9 | — | 2 lb. | 1 oz. nearly |
| 2 to 3 | — | 3 | — | 2 | $4\frac{1}{3}$ |
| 3 to 4 | — | 5 | — | 2 | $6\frac{1}{5}$ |
| 4 to 5 | — | 3 | — | 2 | 7 |
| 5 to 10 | — | 9 | — | 2 | $8\frac{4}{9}$ |
| 10 to 15 | — | 14 | — | 2 | $12\frac{4}{14}$ |
| 15 to 20 | — | 3 | — | 2 | 13 |
| 20 to 30 | — | 19 | — | 2 | $12\frac{14}{19}$ |
| 30 to 40 | — | 22 | — | 2 | $13\frac{14}{22}$ |
| 40 to 50 | — | 29 | — | 2 | $14\frac{20}{29}$ |
| 50 to 60 | — | 35 | — | 2 | $13\frac{2}{35}$ |
| 60 to 70 | — | 42 | — | 2 | $11\frac{34}{42}$ |
| 70 and upwards | | 44 | — | 2 | $10\frac{5}{44}$. |

"The inference from this table is, that the average weight of the brain goes on increasing from one year old to 20; between 20 and 30 there is a slight decrease in the average; afterwards it increases, and arrives at a maximum between 40 and 50; after 50 to old age, the brain gradually decreases in weight."

As, during the greater part of the progress of most cases of organic diseases of the brain, the most important symptoms observed are of short duration, and often at long intervals, it is quite obvious that these symptoms cannot depend *solely* on the permanent lesions of texture which appear on dissection. The simplest view of their connexion with the organic lesions is, that the latter are great and permanent *predisposing* causes of the diseased actions on which they immediately depend. The *exciting* cause of the occasional urgent symptoms is most generally to be sought in a disordered state of the circulation in the head; and here we perceive the close connexion of these organic diseases with the next great genus of diseases of the brain.

III. Of the Apoplectic Cases, Dr. Abercrombie, after enumerating the most common premonitory symptoms, makes three general divisions: 1. Those which are primarily apoplectic; i. e. where the patient becomes comatose suddenly. 2. Those in which the patient is first seized with pain of head, sickness, and faintness, and often falls down, but almost immediately recovers himself, and then passes gradually into fatal coma. 3. Those in which the first attack is of paralysis, more or less general, without stupor, or with slight and transient stupor only.

In the first class, in which the patient is comatose from the first decided attack, it is singular that he does not include a single example where extravasation of blood had occurred; all the cases he has seen were either successfully treated, or, if fatal, showed on dissection either the serous effusion of which we have already spoken, or no morbid appearance at all; in which last case he uses the term Simple Apoplexy. This appears to be more frequent at or before the middle period of life than in advanced life; it is often preceded by indications of a disordered state of the circulation, and Dr. Abercrombie seems to consider it as always dependent on that cause; but in some cases we think it must be admitted that no such indication can be observed. Of the deranged state of the circulation which would seem to be the sole cause of apoplexy in such cases, and a great part of the cause of urgent affections of the head in many other cases, we shall say a few words presently.

We must observe, however, that there are certainly cases of apoplexy from extravasation of blood, in which the patient becomes insensible suddenly, and coma, if not the first symptom, takes place within a very few minutes after the attack. Indeed, Dr. Abercrombie himself gives two cases of this kind in a subsequent section, (Nos. 116 and 117,) the first of which is remarkable on account of its very short duration; not above five minutes having intervened between the first attack, marked by a loud scream, and the death of the patient. Many of the cases, likewise, of apoplectic extravasation recorded by M. Rostan, in his work "sur le Ramollissement du Cerveau," indicate that the occurrence of any symptoms previously to those which are evidence of extravasation, is by no means so rare as Dr. Abercrombie's experience leads him to conclude it to be; and this certainly accords with our own experience. M. Rostan mentions six cases as occurring "sans signes précurseurs."

2. Dr. Abercrombie has distinguished more accurately, we believe, than any other author a description of cases which, as he says, are not primarily apoplectic, but in which effusion of blood is found more uniformly, and to a greater extent, than in any others; cases where the first attack is of sudden and violent pain, "the patient often starting up and screaming from the violence of it; sometimes he falls down, pale and faint, often with slight convulsion, but very soon recovers from this state. In other cases he does not fall down, but feels great uneasiness in his head, with paleness, sickness, and often vomiting;" the first attack is so far recovered from that the patient often walks home, but the pain of head usually continues, with paleness, coldness, a weak and generally frequent pulse. As the pulse improves and the countenance becomes more natural, then often flushed, the patient becomes more drowsy, and at last sinks into coma, from which he never recovers. The period that intervened between the first attack and the commencement of coma varied, in the different cases he records, from fifteen minutes to three days; indeed, in one case there was reason to believe that fatal coma was connected with an attack of sudden pain, giddiness, and insensibility, a fortnight previously. Generally, however, this interval does not extend beyond a few hours.

Now, on examination after death in such cases, says Dr. Abercrombie, we find none of those varieties and ambiguities which occur in the strictly apoplectic state, but uniform and extensive extravasation of blood. The rupture of a pretty large blood-vessel had caused the first attack of pain, and the symptoms of *concussion*, particularly the faintness and feeble pulse; as this went off, the quantity of blood effused gradually increased, until its pressure on the brain was sufficient to produce coma. "In their whole progress, these cases are strictly analogous to those of extravasation on the surface of the brain from external injury," of which it has been generally held since the time of Pott, that an interval of recovery of sense, after the first shock, and a relapse within a few hours into coma, are the surest indication. We may add that, as the situation of the ruptured vessel in these cases is generally such as to allow the effused blood to spread itself immediately over a considerable surface, the symptoms of the first concussion are in accordance with the doctrine of Dr. Wilson Philip, that any impressions on the nervous system which immediately affect the heart must be made on a large surface of nervous matter.

We quote only a single case of this description, which is remarkable both for its short duration, and for the satisfactory explanation of its rapid progress which the inspection of the body afforded.

"A soldier was seized with giddiness, and fell down: on being raised, he vomited, and complained of violent headach and faintness, but was quite sensible; he was very pale, and his pulse slow and languid. Being carried to the hospital, he asked for cold water, which he swallowed, and seemed relieved from the faintness, but continued very pale. In a few minutes, his eyes became fixed, he *drew deep inspirations*, and in two minutes more he was dead. From the moment of seizure he did not move any of the extremities. On inspection, nothing unusual was discovered in the brain. The vessels of the cerebellum appeared very turgid, and, on removing the cerebellum, a coagulum of about two ounces of blood was found under it, *surrounding the foramen magnum;* which must obviously have pressed on the origin of the par vagum, and so first excited and then suppressed respiration."

3. Dr. Abercrombie's third class (paralytic cases,) consists of those where there has either been no apoplexy, or the apoplectic state has soon passed off, leaving the paralysis, as the more permanent and prominent character of the disease." After remarking on the well-known variety of such cases, both as to the extent and degree of the paralysis, and as to its abatement or permanence, he divides cases of this kind according to the different appearances on dissection, (which, however, can only be guessed at during life,) into the paralytic cases with serous effusion only, or slight morbid appearances; of which we shall say a few words presently;—of those with extravasation of blood to a small extent; of those with ramollissement, and those with other effects of inflammation. He has accurately described some of the varieties of the alterations of sensation which occur in such cases, and some of the curious modifications of the faculty of memory, particularly the loss of the memory of words, while the patient seems to have perfectly distinct ideas of things and their relations; the loss of the memory of one class of words, (most commonly substantive nouns,) while others are remembered; and the loss of all recollection of the right name of a thing, while a new name for it is either invented or transferred from some other object, but, after being once applied, is never again varied. (See p. 277.) It may be added that, besides these very common perversions of the natural associations of ideas in paralytic cases, there is very often observed such a torpor in the succession of thoughts in the mind, that a single object of thought is evidently obtruded on the attention for hours together; or a single emotion, once excited, dwells in the mind for a very unusual time, which is obviously the cause of paralytic persons having so frequently little apparent command over the usual external manifestations of emotion.

Our author very justly insists on one fact in regard to palsy, on which, in the absence of any other indications of the nature of its cause, we ought always to reflect, viz. that "certain cases of it depend on a cause which is of a temporary nature, and may be very speedily and entirely removed;" some of them being probably connected with some local determination, or other derangement of the circulation, without extravasation or permanent lesion of the cerebral texture; others with extravasations or injuries already of old standing, and the effects of which do not extend far from their own immediate vicinity. We cannot be surprised at this when we reflect on the very great variety of formidable symptoms of affection of the brain which may show themselves in cases of simple concussion, and completely disappear after two or three days, although, as Sir B. Brodie well observes, we can hardly conceive such symptoms to be excited by an injury otherwise than by some imperceptible lesion of the delicate structure of the nervous matter.

In the case of palsy from extravasated blood, it is obvious that the abatement of the symptoms depends on the absorption of the coagulum; and this again depends very much on the condition of the cerebral substance just around the coagulum. When there is much softening around it, the case may be speedily fatal, although the extravasation has been slight. This softening, Dr. Abercrombie says, "seems to arise from a diseased state of the arteries of the part; the same, probably, which generally gives rise to the extravasation:" but we apprehend it very often arises also from the inflammation which the effused blood excites in the portion of brain on which it intrudes, and that subduing this inflammation is one of the main objects of the antiphlogistic remedies employed in recent palsy.

As to the time and mode of the absorption, and the appearances in the brain which may be held to indicate that this process has been going on, there is some discrepancy of statement in the works before us, and evidently considerable variety in nature. It is certain that the length of time that elapses before the coagulum disappears is very various. Dr. Abercrombie has described a case (No. 127,) in which nothing but an empty cyst remained in the brain, to account for a stroke of palsy which had occurred five months before; while, on the other hand, we have the authority of Serres for coagula remaining unabsorbed in the brain for two or even three years. In general the change that occurs seems to be that the coagulum is surrounded by a membranous cyst, which is of a yellowish colour and soon becomes organized, (which we apprehend to be a product of the inflammation it excites in the adjoining portion of brain;) and that, when thus enclosed, it is first decolorized, and then gradually absorbed; the organized cyst remaining generally with some soft bands of lymph crossing its interior, and containing in most instances a little serous fluid, and, although gradually shrinking in size, hardly ever becoming completely obliterated till the patient's death. But, although this seems to be the process that is gone through when the health is most completely restored after the paralytic stroke, yet it is a process evidently capable of being variously modified by any disordered condition of the system; in which case, as has been very distinctly pointed out by

Andral, an effusion of blood may gradually be converted into very various forms of organic disease.

On the treatment of apoplectic cases, with or without paralysis, perhaps the most important observation made by Dr. Abercrombie, and illustrated by a reference to his numerous cases, is "that there are no symptoms which characterize a distinct class of apoplectic affections, which in their nature (if seen from their commencement,) do not admit of bloodletting." He gives, in particular, several cases, "occurring in old and infirm people, which might have been considered as modifications of the disease, not admitting of active treatment, yet under such treatment terminating favorably;" and he reminds us that, "in apoplectic affections, the strength of the pulse is a very uncertain guide; for nothing is more common than to find it, on the first attack of apoplexy, weak, languid, and compressible, and becoming strong and full after the brain has been in some degree relieved by large bloodletting."

It is unnecessary to dwell on the importance of free and repeated purging in apoplectic cases, nor on the use of cold applications, and the other parts of the antiphlogistic remedies and subsequent regimen; but we cannot omit quoting the important observation, that "we have been too much in the habit of believing that paralysis of any considerable standing depends on a fixed and irremediable disease in the brain." We see many "recent cases of it completely carried off in a few days; we see others recover more gradually," but completely, within a few weeks or months; and, in many cases of long-continued palsy, where the patient has died of some other disease, we find no morbid appearance in the brain that seems adequate to account for the diseases; certainly no greater lesion of the structure of the brain than has existed in many other cases, where there was no palsy. Thus we know that there are many examples on record of very sudden recovery even from palsy of old standing.

"A case," says Dr. Abercrombie, "occurred to a friend of mine. A middle-aged man was suddenly attacked with hemiplegia and loss of speech, while using violent exercise: all the usual practice was employed without any improvement for a month; the paralytic limbs then became one day suddenly convulsed, and, when this subsided, the paralysis was gone." . . . "A woman, who had been paralytic from the age of six to forty-four, suddenly recovered the perfect use of her limbs when much terrified during a thunder-storm, and making violent efforts to escape from a chamber where she had been left alone. A man, who had been many years paralytic, recovered in the same manner when his house was on fire; and another, who had been ill six years, recovered suddenly in a sudden paroxysm of anger."*

"Such examples point out a most important principle in regard to paralysis, namely, that cases of it, even of long standing, sometimes depend on a cause which may be removed entirely, and removed almost in an instant;" in fact, we now know that they depend very generally on a cause acting in a part of the nervous system at some distance from those parts of it on which sensation and voluntary motion *immediately* depend; and that, although the influence of the injury done to the brain very often extends downwards to the medulla oblongata and spinal cord, yet it does not do so uniformly, necessarily, nor permanently.

We have not, however, as yet, any remedies on which we can rely,

* Diemerbroeck.

and which we can effectually control, to be used in imitation of the spontaneous beneficial changes which attest this principle. In order that any stimulating remedies may be used with safety, Dr. Abercrombie thinks it necessary that "the system should be at the same time kept in a very low state by spare living and occasional evacuations." He passes several such remedies (among others, strychnine,) in review, but concludes, on the whole, that, in general, as auxiliaries to the natural process of restoration of power, "we can employ nothing better than much dry friction and persevering exercise of the limbs themselves, as soon as they shall have recovered the slightest degree of motion." It may be hoped that more powerful remedies of this class may yet be devised; but it is plain that the main object must always be so to regulate the circulation within the head as to prevent renewals of the apoplectic paroxysm.

We must here say a few words of the speculation of Dr. Kellie, Dr. Abercrombie, and many others, on that peculiarity of the circulation in the head which must certainly be an element in the pathology of all the diseases of the brain,—viz. the circumstance of the circulation being here confined to a cavity which is completely protected from the pressure of the atmosphere, and which is always filled with matters that are nearly incompressible. The natural consequences of this proposition, if strictly true, are, that the cavity of the cranium cannot contain either more or less blood at any one time than at any other, and that no unusual quantity of blood can be either added to it or subtracted from it, without an equal quantity, within the same time, making the opposite movement. That the proposition is true, or very nearly so, appears distinctly from various facts, particularly from the observation of Dr. Kellie, that, in animals bled to death, when the cranium is entire, the vessels of the brain are found well filled, or at least not drained of blood like those of other parts of the body, (excepting in a few cases where the place of the blood that had drained from the vessels seemed to be taken by a little extra-vascular serum;) but, if an opening is previously made in the cranium with the trephine the vessels of the brain are as completely drained as those of other parts; again, from the observation of Dr. Monro and others, that, after strangulation, the vessels of the brain do not appear peculiarly distended; and lastly, from the observation of Gendrin and Beclard, that, after decapitation, these vessels are found as turgid as usual.

But, while it is easy to be assured of this peculiarity in the circulation within the head, and of its important use in the healthy state, in rendering the pressure on the brain much more uniform than it could otherwise have been, it is not so easy to be certain of the effects it will produce in the different diseased states of the circulation; and we confess ourselves doubtful how far Dr. Abercrombie's speculations in regard to it are well founded.

He endeavours to form a judgment of the effect which will result either from increase or diminution of the impetus with which the blood enters the cranium by the arteries, on the relative quantities of blood in the arteries and veins within the cranium; and supposes that any derangement of that relation will materially derange, or even suspend, the functions of the brain. Without pronouncing definitively on this point, we think a simpler view of the consequences of derangement of the circulation within the head may be taken, requiring only this preliminary postulate, that the natural state of the functions of the nervous system requires that the nervous matter be subjected to a certain degree of *pressure*, and that either increase or diminution of that pressure, if rapidly effected, will injure or suspend these functions. It is plain that, although the *quantity* of blood within the cranium can hardly be altered, the *impetus* with which it enters, and the degree of pressure it exerts on the nervous matters, is liable to much change; and it is well observed by the late Dr. Monro, that, the less compressible we suppose the substance of the brain to be, the more easily we can conceive it to be injured by an excessive impetus of the blood against it.

In the natural and healthy state it is obvious that the circulation may be very much accelerated, and the impetus of the blood by the arteries much increased, as by exercise, full diet, or a moderate quantity of stimulating liquors, without the functions of the brain being impaired; the succession of mental acts being only somewhat accelerated; but in such cases there can be no doubt that the velocity of the flow of blood in the veins, (every where much dependent on the impetus of the blood in the arteries, and particularly so in the cranium, where the whole quantity of blood is so uniform,) is increased just in proportion to the increased impetus by the arteries, and the substance of the brain thereby escapes any morbid pressure. But if, along with the increased impetus, there be any obstruction, general or partial, to the free passage of the blood through, or its free exit from, the vessels of the head, the nervous matters will be generally or partially compressed, and some alteration of its functions may be expected. Now, this obstruction to free circulation may be from various causes; and therefore various causes render an increased impetus of blood in the arteries of the head dangerous. Thus, a diseased state of the vessels themselves, or tumour already existing within the cranium,—an impediment, whether permanent or occasional, to the free descent of the blood along the jugular veins, (as in the hooping cough of children, or in various diseases of the heart and lungs in adults,) becomes a cause of increased pressure, and sometimes of effusion on the brain and of urgent symptoms. Again, the free passage of the blood through the vessels within the cranium, as in all other parts of the body, is in a great measure dependent on causes not yet elucidated, but which are known to act exclusively in the small capillaries. When the causes which in the natural state promote the flow there are deficient, the arterial blood will have a difficulty in making its way, and there will be an increased pressure, and perhaps effusion; and this seems to be what happens in fever; in which case, without admitting the old theory of a spasm of the capillaries, we may safely affirm that there seems to be generally over the body a deficiency of vital action in the capillaries, demanding an increased or more frequent impulse from the heart, in order that the circulation may be maintained.

Again, when the impetus of the blood entering the cranium by the arteries is diminished, if it be done very gradually, the flow by the jugular veins is proportionally retarded; the pressure on the brain remains uniform; and in such a case we know (as, e. g., in the case of a patient dying of enteritis,) that the head may remain perfectly clear, and the senses quite entire, after the pulse has become imperceptible at the wrist. But, when the impetus of arterial blood on the brain is *suddenly* diminished, the mere circumstance of retarded efflux of blood by the veins is inadequate to compensate for that diminution; the pressure previously exerted on the brain and medulla oblongata is sensibly lessened, and more or less of insensibility results. This appears distinctly to be the rationale of the insensibility of syncope from loss of blood, from the erect posture, and from tapping for ascites; and likewise, according to Dr. Kay, of the insensibility of asphyxia; and all these cases appear to be illustrated by the insensibility produced, in the experiments of Magendie, by suddenly drawing off the "cerebro-spinal liquid."

There are a few cases of disease, in which the diminution or extinction of sensibility, and the state of coma, more or less profound, connected with diminution, not increase of the pressure of arterial blood on the brain, are exemplified; and these have attracted a good deal of attention of late years. Dr. Marshall Hall has given to such cases, occurring in children, the title of *hydrocephaloid*, which we think a fair and appropriate one; but we cannot admit his claim to priority in distinguishing and describing them. His words are, "I first gave a cursory sketch of this morbid affection in a little volume of 'Medical Essays' published in 1825. —It has since been briefly noticed by Dr. Abercrombie, in his valuable 'Researches,' &c., published in 1828.—Lastly, Dr. Gooch has treated of this affection in his excellent 'Account of some Diseases peculiar to Women,' published in 1829. . . . These dates will settle the questions of priority and originality." But he does not seem to be aware that the substance of the greater part of Dr. Abercrombie's book was published in the Edinburgh Medical and Surgical Journal as far back as 1818. From the Number for November of that year (vol. xiv. p. 581,) we extract the following passage, which, we happen to know, was quoted and commented on by some of the teachers at Edinburgh, in 1820-21.

"In the end of diseases of exhaustion, patients frequently fall into a state resembling coma a considerable time before death, and while the pulse can still be felt distinctly. I have many times seen children lie for a day or two in this kind of stupor, and recover by wine and nourishment. It is often scarcely to be distinguished from the proper coma which accompanies affections of the brain. It attacks them after some continuance of exhausting diseases, such as tedious and neglected diarrhœa; the patients lie in a state of insensibility, the pupils dilated, the eyes open and insensible, the face pale, and pulse feeble. It may continue for a day or two, and terminate favorably, or it may be fatal. This affection is the only disease that I have seen which corresponds with the *apoplexia ex inanitione* of the older writers."

This is obviously the same affection as is described by Dr. Hall, at p. 66-7 of the work before us. Dr. Abercrombie adds, "I have seen in adults an affection approaching to this, and from the same cause. A man considerably advanced in life, from a neglected diarrhœa fell into a state very much resembling coma; his face pale and collapsed, but his pulse of tolerable strength. An elderly lady, from the same cause, had loss of memory and squinting. Both these cases recovered by wine and spirits." In some such cases we have seen, on dissection, decidedly morbid serous effusion within the cranium, though attended with none of the indications, and preceded by none of the symptoms of inflammation.

Dr. Abercrombie has illustrated this point further in his later work by several cases, in which, after great loss of blood from various causes, when the pulse was frequent and feeble, easily excited, and the countenance pale and exhausted, there was much complaint of headach, throbbing in the head, giddiness, tinnitus aurium, &c. generally attended by palpitation, and much increased by any exertion; and all these symptoms were effectually relieved by the cautious use of wine and of a fuller diet. (P. 306 *et seq.*) The cases described by Dr. Marshall Hall and by Dr. Armstrong, under the title of Reaction after the Loss of Blood (likewise very distinctly described by Rush, in his treatise on Bloodletting,) are of the same general character. How far the symptoms in such cases, which bear so striking a resemblance to those of plethora and increased determination to the head, are to be ascribed to the stagnation of the venous blood and turgescence of the veins, consequent on the diminished influx from the arteries, or how far they may be more directly ascribed to that diminished arterial flow and diminished pressure, it is difficult to judge; but it is plain that another element must enter into the explanation of at least a part of these symptoms,—viz. the increased irritability or mobility, and more rapid (even although they be more feeble) contractions of the heart, which are observed in these circumstances.

After all that has been said of the consequences which appear to result from the peculiarity of the circulation within the cranium, it is still to be borne in mind that, within the cranium itself, the distribution of the blood in the different small vessels is liable to varieties, equally as in other parts of the body, which depend on causes acting on individual portions of the brain or of its membranes, and are exemplified in the case of partial inflammation or in the growth of all kinds of organic disease, and which are compatible with perfect uniformity in the whole quantity of blood entering and leaving the cranium. And, when we speak of the effects either of increased or diminished impetus upon, or of accelerated or retarded transmission of blood through the brain, we must also remember that many of these effects are in a great measure determined by the sound or diseased state of the vessels in different parts of the cranium; the influence of which on the production, not only of extravasation of blood, but of other diseases of the brain, appears from many facts in the volumes before us.

We have already expressed our conviction that the different genera of disease now briefly considered, do by no means exhaust the pathology of the nervous system, and we think it obvious that there are two others, of sufficiently distinct character and sufficient practical importance, to demand a short notice here.

The first is, the case where the functions of the nervous system are deranged, (symptomatically we admit,) not by any alteration of its supply of blood, nor of the nutrition of its texture, but by a vitiated state of the blood which pervades it. We here allude, not merely to the effect of poisons absorbed from the stomach, skin, or lungs, nor to the contagious poisons, also absorbed from without, and "multiplying themselves in the mass of blood," nor to the purulent or other inflammatory effusions which seem certainly to circulate in the blood in the cases of inflamed veins; but especially to the effect of excretions, formed and retained in the blood, instead of being thrown out by the natural outlets. The poisonous nature of all excretions, if retained in the circulating fluids, is indeed a general law, not only of all animals, but of all living beings, and the nervous system of animals seems distinctly to be the part of their frame, on which they exert their chief noxious influence. The most striking example of this kind is the ischuria renalis, where the excretion by the kidneys is suppressed, and the urea formed circulates in the blood; a case which is well known to terminate almost uniformly, as the symptoms produced in an animal by extirpation of the kidneys do, by death in the way of coma. A more common occurrence is the alteration and great diminution of the solid contents of the urine in cases of Bright's disease of the kidneys; a considerable proportion of which cases are also fatal in the way of coma. When death has been produced in either of these ways, we have repeatedly found, on dissection, that the profound coma and spasms preceding it had been unconnected with any serous effusion or perceptible disorganization of the brain, but connected with the appearance of urea in the blood, and in the usual effusions on all the serous surfaces. We repeatedly have seen jaundice terminate fatally in the way of coma, and found on dissection, in every such case, the brain healthy, and bile ducts *pervious and empty ;* and this has been so often remarked by others, (Morgagni, Marsh of Dublin, &c.,) that we can have no doubt of this being the usual state of things in that form of jaundice, and that the *retained* excretion of bile is more decidedly and rapidly poisonous to the nervous system than the *reabsorbed* excretion in the more usual forms of jaundice. And, when we observe the similarity of the delirium, spasms, and coma in such cases, to those of bad typhoid fever; when we consider how all the excretions are altered in that case, and remember that any serous effusion or obvious disorganization in the brain after such fever bears no fixed proportion to the degree of such symptoms, and is often altogether absent when such symptoms have been urgent; we are naturally led to the belief that much of the affection of the brain in the course of fever, and of other epidemic diseases, is to be ascribed to the depraved quality, not to the increased quantity or impetus of the blood. This is a subject, however, the truly scientific investigation of which is only now commencing, and on which it would be premature to speculate farther at present.

The last enquiry on the pathology of the nervous system is one of the most interesting of all, but likewise, hitherto, very imperfectly investigated; viz. What is the nature and extent of the alterations which its functions can undergo in disease, independently of any known or perceptible alteration, either of its structure or of the quantity or quality of the blood which pervades it?

The case of simple apoplexy is one in which not only no effusion capable of injuring the nervous system is perceptible, but no alteration of structure can be detected, and there are certainly cases of that kind in which no indication either of increased or diminished impetus of the blood on the brain, such as might cause distention either of arteries or veins, can be observed. Some cases of epilepsy, or of hysteria nearly akin to epilepsy, appear to be nearly in the same predicament. Cases of concussion, cases of syncope, or even of sudden death from mental emotion, and still more remarkably cases of hysterical coma, all instruct us that the functions of the nervous system may be completely suspended, without any such injury of its texture as can prevent a speedy and complete retention of all its vital powers; and, notwithstanding all that has been written on nervous disorders, the possible extent and varieties of these strictly functional affections of the nervous system (which form a study in themselves, and cannot be treated as the consequence of any known change in the condition of the organs diseased,) still require to be laid down and defined. Dr. Abercrombie has given several fatal cases of paralysis long continued, with pretty violent spasms, (Nos. 154,-5,-6,-7,) in which nothing morbid could be detected after death; and several cases both of spasmodic and paralytic symptoms, (p. 399 *et seq.*) apparently exactly similar to those which depend on organic disease, but of which the ultimate result was so favorable as nearly to set aside that supposition. Cases of neuralgia abating under the use of bark, arsenic, steel, hydrocyanic acid, &c. must be regarded likewise as strictly functional; and the same may be said of chorea, of the whole tribe of hysterical affections, of catalepsy, and of the strange and anomalous disorders commonly called spasmodic, but more properly regarded as perversions of the mental part of the process of voluntary motion, which have been so often observed to be propagated by imitation, and so to become epidemic. It may be said, indeed, that in all these cases there is some disorder of the circulation in the head; but, although this be admitted, it is plain that this disorder must be slight, probably not more than can exist in other cases, without any symptom resulting from it; and that some other condition of the nervous system itself must coexist with it in order to produce these singular effects.

The most curious question that has lately attracted attention, illustrating the degree of alteration of the functions of the nervous system, which may take place independently of any apparent injury of its structure, is that which regards the reality or fictitious nature of the statements we have of the state of somnambulism or "extase," and of the influence of what is termed animal magnetism in exciting it. The most numerous collection of statements on these subjects recently published is that contained in the work of Mr. Colquhoun, to which he has given the fantastic title of Isis Revelata. It was our intention, on commencing this article, to hazard a few observations on the very curious matters contained in this volume, (foreign as we know some of our readers may think them to the legitimate subject of the article,) with the hope rather of placing the subject in a proper point of view, than of enabling our readers to arrive at a positive conclusion in regard to it. Our materials, however, have already run to so great a length that we must postpone this discussion for the present.

